# Supporting relatives when general palliative care is provided at home– a focus group study based on nurses’ experiences

**DOI:** 10.1186/s12904-025-01744-z

**Published:** 2025-04-21

**Authors:** Elina Mikaelsson Midlöv, Susann Porter, Therese Sterner, Katarina Sjögren Forss, Terese Lindberg

**Affiliations:** 1 Blekinge Institute of Technology, Faculty of Engineering, Department of Health, Valhallavägen 1, Karlskrona, 371 79 Sweden; 2 Faculty of Health and Society, Department of Care Science, Malmö University, Malmö, Sweden

**Keywords:** Home care, Nurses, Palliative care, Relatives, Support

## Abstract

**Background:**

Relative’s efforts are essential when palliative care is provided at home and support from healthcare professionals is needed. Despite this, since the support provided varies, relatives may have unmet support needs. Many people receive general palliative care at home rather than specialised care, and nurses play a significant role in supporting relatives. This study aimed to explore registered nurses’ experiences of supporting relatives before and after a patient’s death when general palliative care is provided at home.

**Methods:**

This study used a qualitative explorative design. Data were collected through focus group interviews with 18 registered nurses in home care in Sweden and were analysed using content analysis. The Consolidated Criteria for Reporting Qualitative Research checklist was used for explicit reporting.

**Results:**

The findings are presented in four categories with subcategories: receiving support to provide support, continuously providing understandable information, balancing different needs and building relationships facilitates safety and identifying needs.

**Conclusions:**

Even if registered nurses support relatives to some extent, they rarely reflect on the support they provide and lack structure in providing support both before and after the patient’s death. The findings showed inadequacies in support after the patient’s death, which is also emphasised in previous studies. The findings also showed deficiencies in routines, local guidelines and checklists as well as in training and education on how to support relatives when palliative care is provided at home, thereby risking that relatives’ needs remain unmet. This highlights the need for creating routines and developing detailed local guidelines and checklists on providing support to relatives both before and after the patient’s death.

**Supplementary Information:**

The online version contains supplementary material available at 10.1186/s12904-025-01744-z.

## Background

In palliative care (PC), patients often want to be cared for and die at home [1–4]. Relatives (e.g., family and friends) play an important role in PC at home [5–7], since it often relies on their efforts, and they take great responsibility, face challenges and are at an increased risk of ill health themselves [6, 8–10]. Therefore, it is essential that relatives are supported by healthcare professionals (HCPs). When PC is provided at home, nurses play a significant role and are often the HCPs who care for the patient and their relatives [11], including supporting them [12–13]. The care provided is influenced by, for example, nurses’ knowledge of and attitudes towards PC [14–15], and the support provided by nurses may vary based on their intuition and experience [16]. Nurses may also find it challenging to meet the needs of both patients and relatives, as these are not always correlated [11]. Although support for relatives is a significant part of PC [17–18], relatives involved in PC at home have unmet support needs [16, 19–21] and may not receive the support they need before or after the patient’s death [16, 22–26]. Unmet needs refer to the absence of a desirable, necessary, or useful action taken or a resource provided, for a person to achieve optimal well-being [27] and provides information about lack of support [7]. Support is grounded in human needs, and supportive actions are essential to meet the needs that a person cannot maintain on their own. In PC, the support needs of relatives can change rapidly depending on the progression of the patient’s illness and relatives’ situation [28–29].

At home, nurses and nursing assistants (NAs) spend most time with patients and their relatives and work closely with them [30–31]. Support from HCPs who spend the most time with relatives in the home is important for how relatives perceive their situation, and the purpose of the support is to prevent ill health and promote the ability to cope with difficulties before and after the patient’s death [32]. It’s important that relatives feel participation and confidence in the care, and HCPs need to be responsive to relatives’ needs, such as the ability to absorb information and have counselling [30]. Supporting relatives may involve providing them with assistance in the care and supporting them informatively, emotionally and practically. Emotional support can be provided through counselling, while practical support may involve relief in the home. Education, information, personalised relief and various forms of counselling are essential [33]. Nevertheless, previous studies have shown that relatives’ need for information and effective communication with HCPs are among their most commonly unmet needs [34–38], while various barriers to adequate support for relatives have been reported, such as an underestimation of their needs, a lack of time and feeling unprepared [39–41].

In Sweden, support for relatives varies when PC at home is provided [29], and many people receive general PC rather than specialised PC. Specialised PC applies to a multi-professional team, HCPs with a higher level of education and a main focus on providing PC, while general PC is usually provided by HCPs, such as registered nurses (RNs) and NAs with basic knowledge of PC, without it being their main activity [30]. In general PC at home, the conditions for supporting relatives can be limited and challenging [30] and the support provided can be lacking [24, 42]. To be able to improve support, more information is needed on what support is provided by HCPs to understand what facilitates and hinders supporting relatives [23]. Since previous research has identified that relatives involved in general PC at home have unmet support needs, and nurses have a central role in supporting them, the aim of this study was to explore registered nurses’ experiences of supporting relatives before and after a patient’s death when general palliative care is provided at home.

## Methods

### Design

This study used a qualitative explorative design with inductive content analysis in accordance with Elo and Kyngäs [43]. It was based on the focus group methodology described by Krueger and Casey [44], which emphasises participants’ shared experiences and shifts the power dynamic from the researcher to the participants, who are positioned as experts in the subject in focus and influence group interactions. The Consolidated Criteria for Reporting Qualitative Research checklist [45] was followed for explicit reporting to improve transparency and the study’s quality (Additional file 1).

### Participants and setting

In Sweden, municipalities are responsible for healthcare services at home, including general PC, and care is provided by RNs, NAs, occupational therapists and physiotherapists, among others [46]. RNs have a leadership role in home care, and since municipalities do not employ doctors, RNs are highly medically responsible and contact regional primary care doctors when needed. In this study, the participants were from urban (*n* = 2) and rural (*n* = 2) municipalities in southern Sweden.

The inclusion criteria for the study participants were as follows: RNs in home care who had experience providing general PC at home. RNs working in nursing homes were excluded. For representativeness in the sample, variations were sought in terms of gender, age, work experience and, if possible, education levels. The sample consisted of 18 RNs (15 women and 3 men) aged 31–63 years. The participants had worked in home care for between 1.5 and 16 years, and several were specialised nurses, but not in PC. See Table 1 for the characteristics of the participants.

**Table 1:** Overview of the participants’ characteristics

Participants(P) (*n* = 18)	Gender	Age	Yearsas RN	Yearsin home care	Nurse education	Specific education in PC	Municipality (*n* = 4; 1 and 4 = urban, 2 and 3 = rural)
FGI 1 (*n* = 5)							
P1	Female	43	18	14	District nurse		1
P2	Female	54	15	10	Geriatric nurse		1
P3	Female	36	4	4	District nurse		1
P4	Female	52	21	3	District nurse		1
P5	Female	53	10	10	Geriatric nurse		1
FGI 2 (*n* = 4)							
P1	Male	39	16	1.5	Anaesthesia nurse		1
P2	Male	31	4	1	Registered nurse		1
P3	Female	55	32	1	District nurse		1
P4	Female	40	15	8	District nurse		1
FGI 3 (*n* = 4)							
P1	Female	53	28	10	Registered nurse		3
P2	Female	56	35	2	Ambulance nurse		3
P3	Female	47	20	5	District nurse, intensive care nurse		3
P4	Female	63	20	15	Registered nurse	Course in PC (8 ECTS)	3
FGI 4 (*n* = 5)							
P1	Male	50	18	15	Registered nurse		3
P2	Female	46	19	10	District nurse	Course in PC (7.5 ECTS), ongoing specialist nurse in PC education	4
P3	Female	48	16	8	District nurse		1
P4	Female	43	17	3.5	Theatre nurse		2
P5	Female	45	17	16	Geriatric nurse		2

### Recruitment and data collection

To access participants, heads of departments involved in home care were contacted for consent to conduct the study and to recruit RNs from their practices. Due to difficulties recruiting RNs in this way, along with purposive sampling, snowball sampling was used [47]. RNs who had received written information about the study from heads of the department or the researchers informed other RNs involved in home care about the study and asked for their consent for the researchers to contact them. To recruit participants, emails containing study information were sent to the RNs. Those interested in participating contacted the first author via e-mail. These RNs received further study information and were given the opportunity to ask questions and provided written consent to participate.

Data were collected during spring 2024 through focus group interviews (FGIs), with 4–5 participants in each focus group [44]. In three focus groups, the participants were from the same municipality but did not work in the same area, while in the fourth, the participants were from different municipalities. A semi-structured interview guide was developed by the researchers based on the aim of the study and was used during the FGIs to ensure that certain topics were covered and to obtain detailed narratives (see Table 2). A pilot interview was held by the first and last authors to evaluate the questions. No changes were needed because the answers that emerged responded to the aim of the study, and this interview was therefore included in the study. Two FGIs were conducted in neutral rooms at the RNs’ workplaces, and two FGIs were conducted digitally using the Microsoft Teams digital platform. The first author acted as a moderator during the FGIs and the last author as an assistant moderator. The FGIs started as open dialogues in which the participants were encouraged to talk freely with each other about their experiences of supporting relatives. The semi-structured interview guide was used as a reminder to ensure that certain topics were covered, and follow-up questions were asked to encourage deepening. The participating RNs also asked each other questions to develop the discussions. During the FGIs, supporting notes were written regarding the impressions and thoughts during and directly after the interviews. Each interview lasted between 55 and 65 min, with an average time of 60 min. After the fourth FGI, no new information emerged.

**Table 2:** Description of the questions in the interview guide

Questions in the interview guide
Tell us about the support you provide to relatives during the patient’s illness at home
Tell us what you think works well and less well with the support for relatives during the patient’s illness
Tell us if you think there is anything that needs to be improved regarding support for relatives during the patient’s illness
Tell us about the support you provide to relatives after the patient’s death
Tell us what you think works well and less well with the support for relatives after the patient’s death
Tell us if you think there is anything that needs to be improved regarding support for relatives after the patient’s death

### Data analysis

All interviews were audio-recorded, and the first author transcribed the material verbatim. The analysis was based on 78 pages of transcribed interview material in a Microsoft Word document. The transcripts were analysed according to the recommendations of Elo and Kyngäs [43] for inductive qualitative content analysis, meaning that the categories were derived from the data. The analysis process involved three main phases: preparation, organising and reporting [43]. In the preparation phase, the recorded interviews were listened to several times, and the transcribed material was then read through multiple times to decide what to analyse and to select the unit of analysis. The written material was then read through again for data immersion and to obtain a sense of the whole. In the next phase, data were organised, starting with open coding; notes and headings were written in text, while the data were reread through several times. The headings were then compiled into coding sheets, and potential categories were generated. The list of categories was grouped together, based on similarities or dissimilarities, into broader higher-order categories to reduce the number of categories. The final categories and subcategories were generated through abstraction [43]. All the authors participated in the analysis. The first and last authors made a preliminary analysis, which was shared with the other authors and discussed further. Dialogues were conducted over time until consensus was reached, and sufficient theoretical saturation was considered to have been reached when no new insights or information emerged to further develop the results. See Table 3 for an overview of the categories and subcategories.

**Table 3:** RNs’ experiences of supporting relatives at home

Categories	Subcategories
Receiving support to provide support	Collaborating with others to provide support
	Providing training to others to give support
	Requesting access to local guidelines and checklists to improve support
Continuously providing understandable information	
Balancing different needs	Meeting different needs during the process
	Taking cultural differences into account
	Having only one chance
Building relationships facilitates safety and identifying needs	

## Results

The findings are presented within four categories with subcategories: receiving support to provide support, continuously providing understandable information, balancing different needs and building relationships facilitates safety and identifying needs.

### Receiving support to provide support

This category describes the need for collaboration to support relatives when general PC is provided at home. RNs play a central role in the team and may need support, but they must also support other HCPs in the home to provide support to relatives.

### Collaborating with others to provide support

The RNs emphasised the need for colleague collaboration to support relatives at home. Having the opportunity, when needed, for two RNs was seen as crucial for handling complex situations and focusing on relatives. However, time and staff availability were insufficient, especially during evenings, nights and weekends. Managers and leadership were described as impacting support for relatives through their control over staff availability and scheduling.

*It’s also important to state the need for two RNs… to care for both relatives and the patient—that you may need support from your colleagues.* (P5, FGI 4)

The RNs stated that they had a central role in the team in supporting relatives by acting as team conveners, for example, coordinating assessments and counselling with doctors. They also relieved relatives’ burdens by coordinating with other HCPs and providing one point of contact for relatives. RNs offer 24/7 phone access, which is an especially important support during evenings, nights and weekends and for distant families. The RNs highlighted the need for teamwork with other professions to support relatives, noting deficiencies and a desire for better collaboration regarding such support. They suggested involving NAs in counselling calls held by doctors in which RNs participate, as well as planning meetings, since NAs spend the most time in the home. Close collaboration with NAs was described as an important part of supporting relatives, although the RNs emphasised the need for better team collaboration to understand how NAs support relatives.

*When I’ve been on home visits with the doctor*,* I’ve always wished*,* or told the NAs*,* that they are welcome to join*,* but it’s extremely rare that they have time*,*… and that’s a shame.* (P4, FGI 3)

The RNs described offering practical support to relatives at home through team collaboration, including care relief, patient monitoring and providing aids to adapt the home and facilitate for relatives. However, they noted the absence of counsellors in their teams —a role that they often took on themselves to support relatives. RNs offer emotional support by listening, being present, and counselling but face challenges in identifying and meeting these needs. Therefore, RNs sometimes contact priests or deacons for additional emotional support and counselling. The RNs emphasised the importance of contacting priests or deacons more often, although many were not aware of this possibility.

*Counsellors—I think they should be more accessible to relatives. Relatives must then visit the health centre to get support*,* but they have to spend time waiting when they need the support here and now. It could be much better.* (P3, FGI 4)

The RNs emphasised the need for support from colleagues with PC expertise. Some municipalities offer PC team consultations, but only during the day. They also highlighted the need for support groups for relatives in PC; however, these were not often available, and the RNs themselves were often unaware if these support groups existed in their municipalities.

In addition, the RNs described the importance of team collaboration to support relatives after the patient’s death, noting that funeral homes provide important support. RNs support relatives by providing practical information about what happens next and by contacting funeral homes, although practices vary by municipality. RNs also inform other HCPs about the death and when supplies should be collected from the home, relieving relatives of such tasks, although they sometimes collect medicines and supplies themselves just to check on relatives. Deficiencies described in team collaboration after the patient’s death included contacting doctors and offering professional counselling support. Despite recognising the need for relatives to talk to a doctor or get professional counselling support, the RNs often struggled to provide or arrange it, which led to relatives not being informed about it. Some RNs mentioned contacting the Church to support relatives also after the patient’s death, although many never use this option.

*At some point afterwards*,* I contacted the Swedish Church for a relative. I think we’re bad at thinking about this*,* but it’s also an opportunity we have. Because even if you’re not a Christian or a believer*,* priests still have very good experience in grief processing and dealing with it. And I know that they came home to this wife… You can initiate such contact. They’re also very accessible in a way that we cannot provide.* (P4, FGI 2)

### Providing training to others to give support

RNs work to empower and train other professionals to support relatives at home, focusing on NAs, who spend the most time there. However, the RNs described that NAs often lack the necessary knowledge to provide PC at home and support relatives.

*The level of knowledge of NAs is quite low. There are very few certified NAs. There are many very young people who have never even seen an old person*,* let alone a dying person*,* so there is a lot to work with. Then there are those who are super-talented*,* but it takes a lot of knowledge.… And since more and more people are dying at home*,* they are sicker and there are complex situations*,* and so on*,* there is a huge gap between the staff who will take care of the patient and relatives.* (P2, FGI 4)

The RNs described NAs avoiding home visits because they felt a lack of control over the situation. To improve NAs’ competence, RNs sometimes provide training and education, and some municipalities offer support from more experienced staff. However, many of the RNs emphasised that in their municipalities, there is no possibility of such support, training or education. In addition, all the RNs stated that the training and education given to NAs on PC was minimal and never covered supporting relatives, but they emphasised the importance of NAs feeling confident in supporting relatives because it facilitates their work in this regard.

*It’s super-important that NAs feel safe in their work environment.… The safer they are*,* the easier it is for us to work*,* too. Because if you have anxious staff*,* you have anxious relatives.* (P1, FGI 3)

### Requesting access to local guidelines and checklists to improve support

The RNs found it difficult to define support, since it varies widely. RNs focus on the patient and rarely discuss or think about support for relatives. There is no general information, plan or routine for supporting relatives, which the RNs believed should exist.

*Relatives are not informed about what kind of support they can get*,* since there is nothing. There’s no plan or routine for what kind of support is provided.… No*,* there’s no package; this and this is what we can offer you.… And maybe it’s a deficiency that we don’t have this.* (P4, FGI 2)

Furthermore, the RNs asked for local guidelines and checklists regarding support for relatives, since these were completely lacking in their organisations. They believed that these tools would improve support, making relatives feel more informed, listened to and involved in care.

*That you have some structure to start from so that it becomes easier—a checklist so that we do not miss important parts.* (P1, FGI 1)

After the patient’s death, RNs do not reflect on the support given to relatives. They assist with the preparation of the deceased and converse with relatives at the time of death, but their involvement is then over. The deceased patient is no longer registered in home care and, therefore, neither are their relatives. Even after the patient’s death, there are no routines, local guidelines, checklists or information materials regarding support to relatives, which need review, according to RNs. However, the RNs described having guidelines for the counselling call with relatives a period of time after the patient’s death, which is the only planned support.

*We do not provide any planned support after the death*,* beyond one counselling call.* (P2, FGI 2)

### Continuously providing understandable information

The RNs felt that many relatives lack information and emphasised their role in identifying and meeting these information requirements by listening to understand the relatives’ needs and clarifying information from other HCPs. RNs provide continuous, concrete information to relatives, balancing honesty with hope. Informative support needs vary and must be assessed individually by RNs. The timing of RNs’ involvement at home affects their ability to assess and meet these needs; late involvement makes it harder to know what information relatives have received and understood.

*But relatives don’t absorb it. You think they do; they nod and go along. Then you go on the next visit and realise that no*,* they haven’t; it wasn’t perceived that way at all.… I think that becomes a little clearer*,* especially in home care*,* where you may have younger patients and so on. It’s difficult to accept*,*… so it’s a challenge to get relatives on board as well.* (P4, FGI 4)

The RNs emphasised the need to repeatedly provide timely information to relatives and ensure their understanding by repeating or rephrasing information. Justifying and clarifying actions and what is happening were seen as crucial. Informing relatives about the patient’s physical changes was described as helping the relatives feel more prepared and secure. Written information complements oral information, and documenting relevant information in medical records was emphasised to support relatives, especially for evening, night and weekend shifts when covering larger areas and unfamiliar patients. The RNs also suggested having a binder with relevant information at home for all staff to access as part of supporting relatives.

*If you come to someone you don’t know—for example*,* in the evening or at the weekend—you really must rely on the documentation.… What is documented? What has already been dealt with? What is the plan?… You rely a lot on documentation.* (P1, FGI 1)

The RNs emphasised the need for continuous information about the patients’ disease progression. An important part of informative support is to receive recurring counselling calls from doctors to help relatives understand that the patient is dying and to inform them about expected events as well as the aims and actions of symptom management and treatments. This was described as setting the foundation for relatives’ expectations. However, the RNs stated that these counselling calls often occurre too late, leaving relatives without the necessary informative support, sometimes depending on difficulties in arranging a time when all participants could attend at home.

*Counselling calls with doctors about the patient nearing death sometimes come too late. It’s difficult to get all people together at the same time.* (P3, FGI 1)

Some RNs stated that they inform relatives about the support and team roles available, including home relief and helpful aids. Information on the cash benefit for caring for relatives is part of the informative support, though not everyone receives it, often depending on the type of relative. For example, the RNs did not think of informing a friend to the same extent as a partner or child.

*Often*,* you don’t know how the care organisation works*,* so then you may have to tell them about it and who is in the organisation.* (P3, FGI4)

In addition, the RNs described the importance of providing information to relatives after the patient’s death. RNs inform relatives that they can reach out later with questions as part of their support. Also, after the death, written information complements oral information, and an information brochure about post-death procedures is provided to relatives.

### Balancing different needs

This category describes how RNs balance different needs when providing general PC at home and supporting relatives. They need to consider the support needed by both relatives and the patient, which may differ. They also need to identify who needs what support and when, take cultural differences into account and make time for relatives, since they only have one chance in these situations.

### Meeting different needs during the process

The RNs described finding it challenging to identify and meet the varying support needs of relatives, especially if multiple relatives or when their needs are not expressed. Support may not be provided if relatives’ needs are not demonstrated, even if needed. RNs need to assess and address varying support needs during the care at home, which change over time and depend on the relative’s condition. The RNs also described difficulties in meeting and balancing the differing needs of patients and relatives at home. Since the RNs primarily meet the patient’s needs, they described this as sometimes leading to the perception that support for relatives is lacking.

*It was only a while ago that we were almost physically removed from the patient*,* who was in pain and had anxiety*,* when relatives did not allow us*,* under any circumstances*,* to give morphine and midazolam. And it’s also difficult because if you were on a ward*,* then you could say*,* “Now you can go out and we’ll take care of the patient.” But when you’re at someone’s home and the patient is living there with their relative*,* then you can’t eject someone from their own home. So it’s a challenge.* (P2, FGI 4)

The RNs also found it challenging to support relatives who are minors at home since they feel less equipped to do this compared to supporting adults. The type of relative may also affect the support offered; for example, supporting a friend was seen as a responsibility different from supporting a partner or child. However, the RNs emphasised the importance of identifying which relatives need the most support but considered it difficult to assess.

*It may be a daughter who hasn’t had contact with her mother*,* but on paper*,* a daughter may outweigh a neighbour. But you may have had a relationship with your neighbour for most of your life.… Once the daughter has come home*,* the neighbour may not be as available*,* but the neighbour has been there for 25 years and has helped. I can think of that.… And then you turn to the wrong person.… So it’s like blood is thicker than water in some way. This becomes a challenge. Then there may be relatives in more need of support but who are not counted as relatives in the same way.* (P1 and P4, FGI 1)

At home, relatives need to talk about the situation, but the RNs described that they could not always meet this need. Relatives often require counselling without the patient present, and the RNs described situations in which relatives would meet them outside to talk before entering the house where the patient is or when relatives ran after them when they were leaving. They stated that they face challenges in finding a time and a place for these conversations. They also emphasised the importance of helping relatives raise uncomfortable issues.

*Discuss things that are uncomfortable to discuss*,*… questions that you might want to ask as a relative*,* but you don’t dare;… it becomes uncomfortable. How long has she left to live? Is this normal? She’s changed and become more irritable; why now?—or whatever it might be.… Because there’s a lot they don’t know.* (P1, FGI 1)

The RNs also highlighted the heavy responsibility of relatives when PC is provided at home and the need to assess how long they can manage. Regularly checking whether relatives are comfortable with the care being provided at home was described as crucial, as they might not speak up. Struggling or unwilling relatives may feel guilt and need support. Continuous assessment of relatives’ willingness and ability to provide care at home was described as essential, as was supporting them in coping with the situation, especially when the patient’s condition was changing. Without adequate support, PC at home may not be feasible, so the RNs emphasised timely and needs-based support for relatives.

*When they are close to breaking point and they can’t really cope with the responsibility that has been imposed on them*,* then it is difficult to relieve it*,* or it is not possible.* (P1, FGI 2)

The RNs described the need to address varying support needs among relatives, even after the patient’s death. They described that first when the patient dies, the focus shifts to supporting relatives. Support is facilitated if the patient dies during the day, allowing the RN in charge to be present. RNs try to stay as long as needed, despite this leading to stress regarding other tasks. If the patient dies at night or on weekends, support for relatives may be impaired. The RNs then stated the importance of the RN in charge contacting relatives when back on duty to check on them. Support after death was described as varying with the age of the deceased. It is easier to support the relatives of an older patient, whose death can be seen as more natural. Supporting the relatives of younger patients was described as more challenging due to its perceived unnaturalness, which may require different support efforts.

### Taking cultural differences into account

The RNs described the importance of taking cultural differences into account when supporting relatives during PC at home. RNs face challenges with language barriers even with interpreters and digital translation aids, making it hard to assess and provide support, since depth is never reached in conversations. Cultural differences in conveying information were described as problematic when supporting relatives, such as when relatives want to control what the patient is told. The RNs described challenges at home when faced with, for example, political symbols or values different from their own but emphasised the need to remain professional and supportive.

*At home*,* it’s not always easy*,* so to speak. There can be cultural stuff; there can be political stuff. You enter the home and see political symbols that are so very foreign*,* like on the fridge door*,* and you start to wonder what’s going on here*,* but you must try to let it go. But*,* of course*,* it can affect.* (P1, FGI 4)

In addition, after the patient’s death, taking cultural differences into account remains crucial in supporting relatives. The RNs stressed the importance of asking relatives about what is important to them. Several RNs stated the importance of taking cultural differences into account and that this should be included in guidelines and checklists.

*We had prepared the patient*,* taken out a small flower and then*,* as we do*,* opened a window. It was a disaster because then you’d let the soul out*,* and the soul wouldn’t find its way back to the body to be able to move on.… And that’s the kind of thing that can ruin the lives of relatives.… It is very important that we ask about such things beforehand.* (P1, FGI 1)

### Having only one chance

The RNs described having only one chance in these situations to support relatives when PC is provided at home and emphasised the importance of listening to, including and facilitating relatives to help them feel safe and involved in decision-making.

*You have only one chance.… Relatives have all their antennae out; everything you say is weighed on a golden scale somehow.… You really must be—you have only that one chance*,* so that you really think.* (P1, FGI 4)

The RNs described the importance of RNs and NAs supporting relatives and patients in achieving their goals and doing things together before the patient dies, supporting them to be able and dare to do so before it’s too late.

*There are some needs they want to achieve before they end their lives that are important to be attentive to.… We have a lot of imagination*,* I think*,* in home care*,* and we should be able to assist with that thing. If the relatives and the patient want to do something together*,* [like] they want to join a swimming practice*,* maybe with their children or grandchildren*,* or something else*,* then we must be able to be there as support so that they dare to do it. I think that’s important.* (P5, FGI 4)

Because there is only one chance in these situations, the RNs emphasised the importance of making time for relatives to feel supported. Although PC is prioritised over other tasks in home care, the RNs described that they often feel a lack of time to meet relatives’ support needs and wished they could do more. They emphasised that more time should be allocated to PC at home, as many relatives need extensive support, regular feedback and home visits. However, no extra time is provided, regardless of how many patients receive PC at home.

*You always prioritise PC; that’s how it is. Then*,* you must run all the faster when you get out of there. But you really try to take the time it requires.* (P3, FGI 3)

### Building relationships facilitates safety and identifying needs

The RNs described often developing close relationships with patients and relatives during PC at home, making it easier to assess their support needs. However, building relationships takes time, so short involvement at home complicates the identification of needs. Longer involvement at home facilitates RNs in supporting relatives and also supports them in utilising their own resources. Challenges were described in supporting new relatives who arrive home late in the palliative phase, as there is too little time to establish relationships. These relatives often have many questions, want a lot of information and have high expectations, which makes it difficult for RNs to provide effective support and manage expectations.

*If you have had a close relationship with the patient and their relatives for a long time*,*… it’s usually easier to see the need for support.* (P5, FGI 4)

The RNs described that it is easier to support relatives with familiar and continuous staff, which creates safety for both relatives and staff and time to establish relationships. This was also described as helping relatives open up about their needs. Experienced staff were emphasised as crucial for safety. The RNs also stated that relatives should be able to change staff if they feel unsafe with them, as feeling safe with staff at home is important support for relatives. Also, regular daytime visits by the RN in charge were described as important; these reassure relatives and reduce support needs in the evenings and weekends, since meeting relatives’ needs can then be challenging.

*Continuity: it provides safety. (P3*,* FGI 2)*

Support for relatives after the patient’s death was also described depending on the relationship. Finding closure with the staff members who have spent most time in the home was described as important, but there is a lack of closure with NAs.

*The NAs do not have any form of closure with relatives*,* like what we have through a counselling call or when we pick up stuff.… But they ask for it. They also want time to call relatives; they may have been there every day for several years. So*,* it’s probably important*,* not only for relatives but also for NAs who have been there a lot.* (P2, FGI 4)

After the patient’s death, the importance of contacting relatives to conduct a counselling call was described and emphasised as a key part of post-death support for relatives. The RN in charge is responsible for the counselling call. This call, offered 6–8 weeks after the death, are based on relatives’ needs and were described as providing closure for both relatives and RNs. It is usually a single call, unless more are requested by relatives, and are made by phone or by visiting the home, depending on relatives’ wishes and where they live. The RNs emphasised the importance of this call for both themselves and relatives and suggested creating routines to address issues raised during these calls to improve support for relatives at home, which is not currently done.

*It’s also a learning opportunity to look back on a period of care together as a team. And then it’s not only a counselling call but also a form of evaluation: what did we do well and what could have been better?* (P2, FGI 3)

## Discussion

The findings from this study show the need for collaboration and that RNs not only support others but also need support themselves to be able to give relatives support. Furthermore, the findings show inadequacies in support, especially after the patient’s death, as well as a lack of knowledge about support for relatives and the absence of routines, local guidelines and checklists.

The RNs described that support from management and leadership was important to support relatives, as they are responsible for time and resources. This is also evident in previous studies, and Hoffstädt et al. [23] have emphasised the need for organisational support to provide the necessary time and resources and to train and educate HCPs. Other studies have also stated that the organisation’s administrative system is a restriction, since there is usually no documentation of relatives’ needs separately from the patients’ needs. Relatives, as well as their support needs, are not visible in administrative systems and may not receive the attention they need [16, 48]; therefore, the needs of relatives need to be assessed separately from those of patients [48]. This becomes particularly important for supporting relatives after the patient’s death. The RNs in this study expressed challenges in that once the patient is deceased, they are no longer registered in home care and, thus, neither are the relatives. This complicated their work in supporting relatives after the patient’s death.

Furthermore, the RNs described the importance of collaboration with NAs when PC is provided at home. Despite describing NAs as key staff, which is also evident in other studies [31, 49], the RNs were unaware of how they support relatives and noted a lack of knowledge about PC and support for relatives among NAs. Several RNs in this study had specialised education, but only two had specific education in PC, and even if the findings showed that RNs sometimes seem to provide competence-enhancing education and training to NAs, most of this is not about PC and never about support for relatives in PC at home. According to the National Board of Health and Welfare [32], organisations need to offer training and education to HCPs to meet the support needs of patients and relatives in PC. Their evaluation of PC in Sweden from 2016 [29] showed ongoing shortcomings in the provision of training and education on PC for HCPs and support for relatives and the work with supporting relatives needs to be reviewed. Thus, although support for relatives is a central part of PC, the findings of this study show that HCPs’ training and education in PC is rarely prioritised, and even less so in supporting relatives. In Sweden, RNs should initiate and lead educational efforts both within the profession and in interprofessional teams [50]. Although there is an organisational responsibility to prioritise training and education for HCPs involved in PC at home, RNs also need to take responsibility for their own continuing training in PC and be involved in ensuring that other HCPs, such as NAs, also receive relevant training. In addition to training and education in PC, other studies [15, 51–52] also emphasise RNs’ attitudes as essential in the provision of PC, and thus also for the support of relatives. RNs’ attitudes towards PC have been shown to be influenced by various factors, such as knowledge, education, previous experiences and resources, and RNs with previous clinical experiences and training and education in PC have a more positive attitude towards the care than those without [15, 51–52].

The findings of this study also show that even if RNs try to support relatives, they rarely reflect on the support they provide them, neither before nor after the patient’s death. This is in line with Hoffstädt et al.’s [23] study, which stated that support for relatives is not systematically integrated into HCPs’ working procedures. Furthermore, Hudson et al. [53] stated that there is no systematic approach regarding support for relatives, and Becqué et al.’s [16] study showed that the support provided by RNs at home is based on intuition and experience rather than on a systematic approach. This unsystematic support risks the support needs of relatives at home being unmet [16, 54], since the support provided may vary based on the RN’s interpretation instead of the relatives’ needs [16]. Previous studies have identified barriers, such as limited knowledge and a lack of resources, that can help in understanding why support for relatives is not part of routine procedures [16, 23]. The findings of this study also show a lack of routines, local guidelines and checklists for supporting relatives in general PC at home, both before and after the patient’s death, which were requested by the RNs. This could be one reason for this unsystematic approach. Although Sweden has national guidelines on PC [30, 55] that include support for relatives, they do not describe in detail how such support can be provided but are more general. Local guidelines on PC may exist in different municipalities, but specific guidelines on how to support relatives are missing. The importance of detailed examples based on various situations and circumstances regarding how to provide support to relatives was previously highlighted in the preparation of the guidelines by Hudson et al. [56]. This needs to be developed in Sweden to improve the possibilities for supporting relatives according to their needs based on a systematic approach. It is important that structured ways of offering support to relatives are applied by RNs and other HCPs to increase the chances of meeting their support needs when general PC is provided at home. An example of this can be to create routines to support relatives and develop local guidelines, and checklists with concrete examples of how support to relatives can be provided during different stages of the process, both before and after the patient’s death. In this study, the RNs expressed that access to local guidelines and checklists could increase the possibility of providing support to relatives based on their needs. They also suggested using issues raised by relatives during counselling calls after the patient’s death to improve support for relatives. This could be a concrete suggestion to address when trying to develop detailed examples in PC guidelines.

Since PC is based on a person-centred approach, support for relatives should also be person-centred. Nysaeter et al. [57] emphasised the need for extensive support from HCPs at home, suggesting a systematic implementation of a person-centred care model and multicomponent interventions by nurses to meet relatives’ need for support. There have been intervention studies on providing support to relatives and studies that have described and evaluated different types of support given to relatives during PC at home; several of these in specialised PC and cancer care [16, 19, 58–64], and the Carer Support Needs Assessment Tool is one example of an instrument developed to identify relatives’ need for support [65]. We suggest that further research should be partly focused on intervention studies in general PC that focus on the competence-enhancing education and training of RNs and NAs, including support for relatives, and ensure how these interventions affect the PC provided and the support for relatives. Rather than conducting extensive education and training programmes, maybe requiring substantial time and resources, short focused workshops, webinars or existing online courses can be used to provide essential training in an efficient way. Additionally, mentorship programmes or professional guidance, where experienced RNs or NAs offer opportunities for practical learning, can also enhance competence in PC and support for relatives. Further work is also needed on creating routines and developing more detailed local guidelines and checklists for supporting relatives in general PC at home in Sweden.

### Methodological considerations

The findings of the study are based on the experiences of 18 participating RNs, which could be considered a small sample for achieving theoretical saturation, but since no new information emerged after the fourth FGI, the data collected were considered sufficient. Because snowball sampling was partially applied, it may have resulted in selection bias that could have influenced the results [47], and since the study only included participants from municipalities in southern Sweden, this may affect transferability, as there are national differences between different municipalities. Also, it is relevant to consider the characteristics of the participants, which may have affected the results. Several of the RNs were 40 years or older and had worked as nurses for several years, even if there was a more even distribution of years worked in home care. The majority were also specialist nurses, although not in PC, and only two had any specific education in PC. Conducting FGIs gave depth to the interviews due to the interaction between the nurses, encouraging them to discuss their experiences and ask each other questions to develop the discussions, which created rich data material. The number of participants in each focus group was considered appropriate because it resulted in creating an atmosphere that generated discussion in which all the RNs could have their say [44]. Although variation among the participants in the focus groups was sought, there may be a risk that some of the focus groups were too homogeneous, possibly resulting in a limited variety of experiences. However, homogeneous groups can lead to more depth in the data. Despite these considerations and limitations, this study provides important knowledge regarding RNs’ experiences of supporting relatives before and after the patient’s death when general PC is provided at home.

## Conclusion

This study showed that even if RNs support relatives to some extent, they rarely reflect on the support they provide in general PC at home, lacking structure in providing support both before and after a patient’s death. The findings showed inadequacies in support after the patient’s death, which is also emphasised in previous studies. The findings also reveal a lack of knowledge about support for relatives and a lack of routines, local guidelines and checklists, which highlights the importance of continued work to improve support for relatives. As previous studies have emphasised, HCPs, such as RNs and NAs, who provide general PC at home need to have relevant knowledge and training in PC and in supporting relatives; otherwise, relatives’ support needs may remain unmet. Since more people will be cared for and die at home, continued work on competence-enhancing education and training in PC and how to support relatives should be a prioritised area in PC. This study also shows the need of creating routines and detailed local guidelines and checklists for supporting relatives both before and after the patient’s death to improve support for relatives. Support for relatives needs to be highlighted and included in the development of general PC, since relatives are important partners when PC is provided at home.

## Electronic supplementary material

Below is the link to the electronic supplementary material.

Supplementary Material 1

## Data Availability

The data that support the findings of this study are not publicly available due to privacy or ethical restrictions but are available on reasonable request from the corresponding author.

## Abbreviations

FGIs: Focus Group Interviews
HCPs: HealthCare Professionals
NAs: Nursing Assistants
PC: Palliative Care
RNs: Registered Nurses
